# Understanding the indirect DNA read-out specificity of I-CreI Meganuclease

**DOI:** 10.1038/s41598-018-28599-0

**Published:** 2018-07-06

**Authors:** Jesús Prieto, Pilar Redondo, Blanca López-Méndez, Marco D’Abramo, Nekane Merino, Francisco J. Blanco, Phillipe Duchateau, Guillermo Montoya, Rafael Molina

**Affiliations:** 1 Structural Biology and Biocomputing Programme, Spanish National Cancer Research Centre (CNIO), Macromolecular Crystallography Group, c/Melchor Fdez. Almagro 3, 28029 Madrid, Spain; 2 Structural Biology and Biocomputing Programme, Spanish National Cancer Research Centre (CNIO), Cell Signalling and Adhesion Group, c/Melchor Fdez. Almagro 3, 28029 Madrid, Spain; 3 Protein Structure & Function Programme, Novo Nordisk Foundation Center for Protein Research, Faculty of Health and Medical Sciences, University of Copenhagen, Blegdamsvej 3B, 2200 Copenhagen, Denmark; 4 Department of Chemistry, University of Rome “La Sapienza”, Piazzale Aldo Moro 5, 00185 Rome, Italy; 5 CIC bioGUNE, Parque Tecnológico de Bizkaia Edificio 800, 48160 Derio, Spain; 6 IKERBASQUE, Basque Foundation for Science, María Díaz de Haro 3, 48013 Bilbao, Spain; 7 CELLECTIS S.A., 8 rue de la Croix Jarry, 75013 Paris, France

## Abstract

The high DNA specificity of homing endonucleases makes them a powerful protein scaffold to engineer enzymes for genome manipulation. Understanding their molecular recognition of DNA is an important prerequisite to generate engineered enzymes able to cleave DNA in specific desired genome sites. Protein-DNA recognition studies have been mostly focused on specific direct contacts between amino acid side chains and bases to redesign the binding interface. However, the important role of indirect readout in the central region of the target DNA of the homing endonuclease I-CreI suggested that indirect readout may play a key role in the redesign of protein-DNA interactions. The sequences of the I-CreI central substrate region, 2NN, along with the adjacent 5NNN, are key for substrate cleavage. Here, we analyse the mechanism of target discrimination at the 5NNN region by the I-CreI protein, revealing its critical role in the location and occupancy of the catalytic metal ions, which is crucial for cleavage. Our data highlight the importance of indirect readout for target DNA cleavage, thus aiding I-CreI engineering when targeting new DNA sequences.

## Introduction

**Figure 1:**
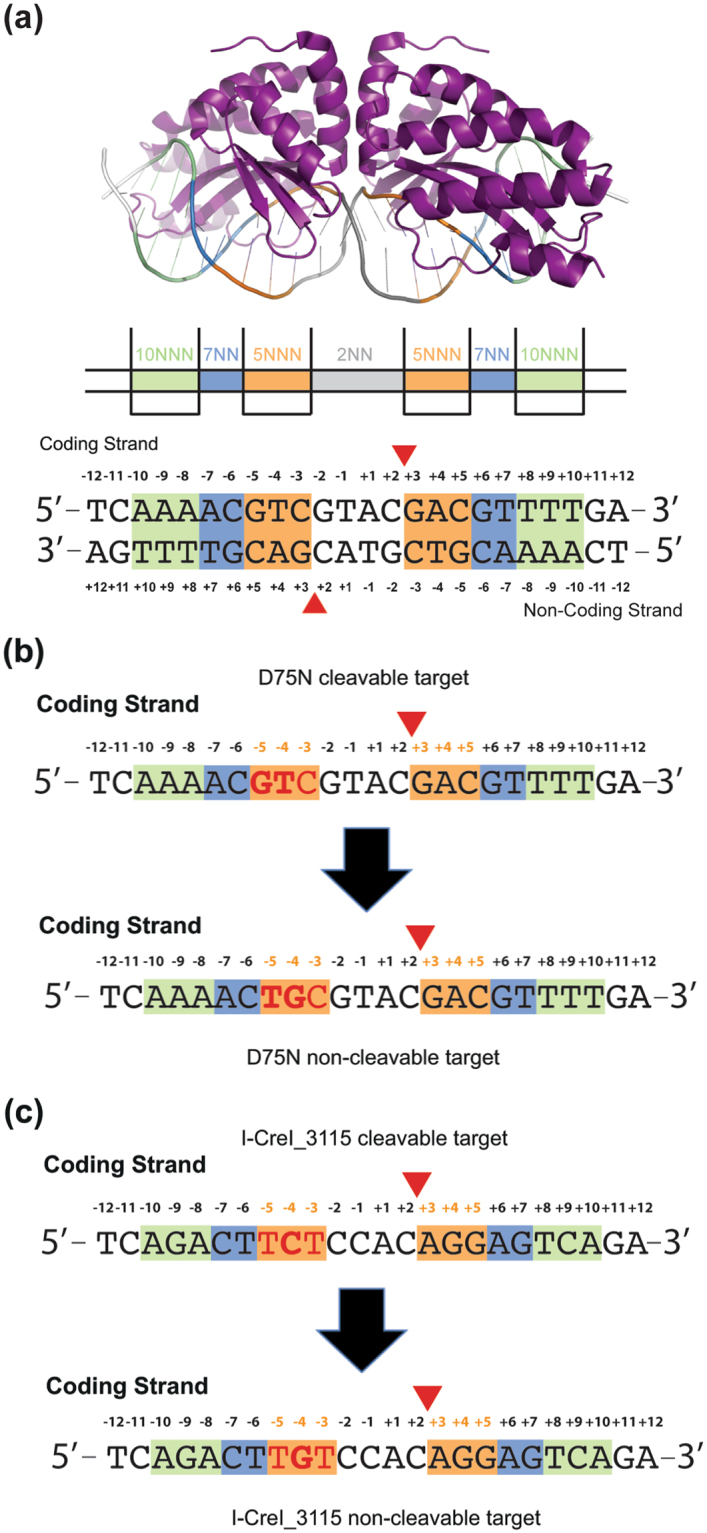
DNA target regions involved in I-CreI_D75N and I-CreI_3115 binding or catalysis. (**a**) The protein–DNA contacts are clustered in three nucleotide regions: 10NNN (±8, ±9, ±10 boxed in green), 7NN (±6, ±7 boxed in blue) and 5NNN (±3, ±4, ±5 boxed in orange). The 2NN region (±2, ±1) is located at the cleavage site (red triangles indicate the scissile P-O bonds). (**b**) Scheme of the design of I-CreI D75N and **(c)** I-CreI_3115 non-cleavable DNA targets based on their corresponding cleavable DNA targets.

Indirect readout in protein-DNA recognition is the mechanism by which the protein achieves partial sequence specificity by detecting structural features on the DNA. Therefore, indirect readout has been proposed to involve contacts mediated by water or other small molecules, as well as distortions of the DNA double helix, so that the protein can distinguish different sequences energetically^1^. Structural analysis of LAGLIDADG homing endonucleases (LHEs) bound to their targets revealed that the central region of the DNA target is kinked, resulting in base twisting and unstacking near the scissile phosphate groups, thus allowing binding and positioning in the active site^2–4^. I-CreI is a homodimeric member of LHEs family, which recognizes and cleaves a 22 bp pseudo-palindromic target (5′- CAAAACGTCGTGAGACAGTTTG -3′). Since different subsets of protein-DNA target contacts may be sufficient to maintain a high degree of sequence-specific homing site recognition and cleavage, some I-CreI-DNA target interactions may be altered and additional changes may be accommodated^5^. Thus, a 24 bp palindromic DNA target is recognized and cleaved by a I-CreI variant (I-CreI D75N) with similar affinity and activity than in the wild-type case^6,7^. Each I-CreI monomer binds its own DNA target region generating the catalytic centre at the dimer interface. This region contains two catalytic aspartic acids (D20, one per each monomer). The aspartic side chains participate in the cleavage of the DNA strands along the minor groove, resulting in the hydrolysis of specific phosphodiester bonds upon the coordination of three divalent metal ions^8^. The structure of I-CreI in complex with its target DNA shows that each monomer establishes direct interactions with the bases^2^, grouped in three boxes called 5NNN located at positions ±3, ±4, ±5; the 7NN located at positions ±6, ±7^9^ and 10NNN located at positions ±8, ±9, ±10^6,10^ (Fig. 1a). The four base pairs (±1 and ±2), called 2NN, containing the scissile phosphodiester bonds, show only one backbone contact between the nucleotide at position −1 (both strands) and K139 (of each I-CreI monomer)^11^. Changes in 2NN significantly affect substrate binding and cleavage. The influence of the central sequence was explained by its topology, showing a mechanism governing target discrimination not based on specific protein–DNA contacts^12^. Thus, the 2NN region affects the active site rearrangement, the proper protein-DNA complex binding and catalytic ion positioning to lead the cleavage.

Here we conduct a study of the impact of some 5NNN target bases composition in catalysis using biochemical, computational and structural analysis. The results suggest a mechanism controlling target discrimination not only based on specific protein-DNA contacts, but on the proper positioning of the catalytic ion. These findings, in line with previous reports^12^ allow us to further rationalize the search for new target sequences in the development of new-engineered homing endonucleases for therapeutic and biotechnological applications.

## Results

### The DNA bases located at the 5NNN region are key for cleavage

**Figure 2:**
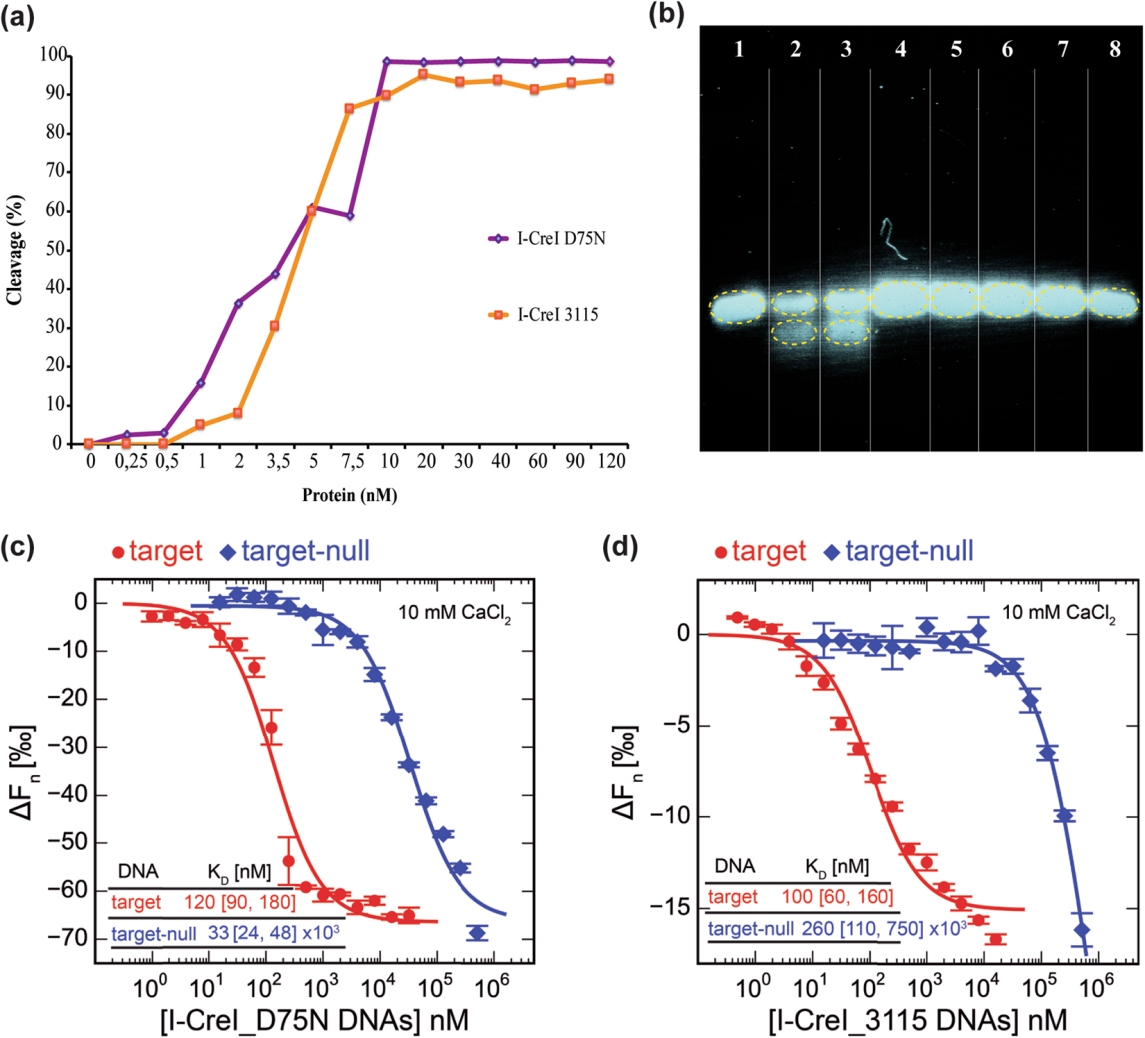
I-CreI *in vitro* cleavage and binding experiments. (**a**) *In vitro* cleavage titration analysis for I-CreI_D75N_target and I-CreI_3115_target using linearized plasmids containing the cleavable target sequences. (**b**) Validating 5NNN non-cleavable target patterns using labelled duplex DNA targets. Lanes: **1**, I-CreI_D75N_target; **2**, I-CreI_D75N_target in the presence of I-CreI_D75N protein; **3**, I-CreI_3115_target in the presence of I-CreI_3115 protein; **4**, I-CreI_3115_target; **5**, I-CreI_D75N_target-null (non-cleavable derivative); **6**, I-CreI_D75N_target-null in the presence of I-CreI_D75N protein; **7**, I-CreI_3115_target-null (non-cleavable derivative) in the presence of I-CreI_3115 protein; **8**, I-CreI_3115_target-null (non-cleavable derivative). (**c**) I-CreI_D75N/I-CreI_D75N_target or target-null binding by microscale thermophoresis (in the presence of 10 mM CaCl_2_). (**d**) I-CreI_3115/I-CreI_3115_target or target-null binding by microscale thermophoresis (in the presence of 10 mM CaCl_2_). The change in thermophoresis is plotted against the concentration of DNA in a logarithmic scale and fitted to a 1:1 binding model to yield the corresponding K_D_s. Numbers in brackets represent the 68.3% confidence interval calculated by ESP (error-surface projection).

Previous studies have pointed out that I-CreI meganuclease has a preference for certain nucleotides at the 5NNN of its target sequence, and that a G in position −4 strongly hampers cleavage^6,9^. To decipher why a G at this position restricts cleavage, we made DNA substitutions in the cleavable DNA target to render it non-cleavable (Fig. 1b,c). For this purpose, we exchanged GT in positions −5 and −4 of the wild type coding strand target (I-CreI_D75N_target, Fig. 1b upper sequence) by TG, generating the non-cleavable target I-CreI_D75N_target-null (Fig. 1b bottom sequence). To broaden our analysis, we also studied the I-CreI heterodimeric variant I-CreI_3115 (Y33G/Q38K/Q44K/R68Y/R70S/D75N/I77Y/I132V-G19S/Y33V/Q38R/S40Q/Q44D/R68A/R70S/D75K/I77R). This mutant was generated using the methodology previously described^6,13,14^, and is able to recognize and cleave the human HBB (Haemoglobin beta subunit) gene (I-CreI_3115_target, Fig. 1c upper sequence) whose mutations cause sickle cell anaemia. C in position −4 of the coding strand target sequence was exchanged to G, generating the non-cleavable target I-CreI_3115_target-null (Fig. 1c bottom sequence). *In vitro* plasmid cleavage assays (Fig. 2a) revealed that both I-CreI_D75N and I-CreI_3115 proteins cleaved their targets with the same efficiency, despite the differences in base composition, amino acid sequences and contacts (see below). Then, to check how a G in position −4 affects target cleavage we performed an *in vitro* cleavage assay using labelled targets, resulting in an inhibition of cleavage when the G is in position −4 position in both DNA targets (Fig. 2b, Supplementary Fig. S1).

### The DNA bases located at the 5NNN region are critical in substrate binding

To analyse whether cleavage differences may arise from changes in binding between the wild type and null DNA targets, we performed MicroScale Thermophoresis (MST) experiments to assess the K_D_ between proteins and target DNAs. Exploratory binding assays using fluorescently labelled proteins or fluorescently labelled DNA targets yielded similar results (Supplementary Fig. S2, Supplementary Table S1). The MST measurements were then performed keeping constant the fluorescently labelled proteins and titrating the corresponding target DNAs in the presence of the non-catalytic cation Ca^2+^ (10 mM). This metal ion allows binding but hinders catalysis^12^. Our results indicate that there are significant differences in the K_D_ between cleavable and non-cleavable targets with both proteins, I-Cre_D75N and I-CreI_3115 (Fig. 2c,d, Supplementary Table S2), providing a possible explanation about cleavage differences at the concentration sampled.

### The 5NNN non-cleavable sequences prevent the central metal positioning in the catalytic site

**Figure 3:**
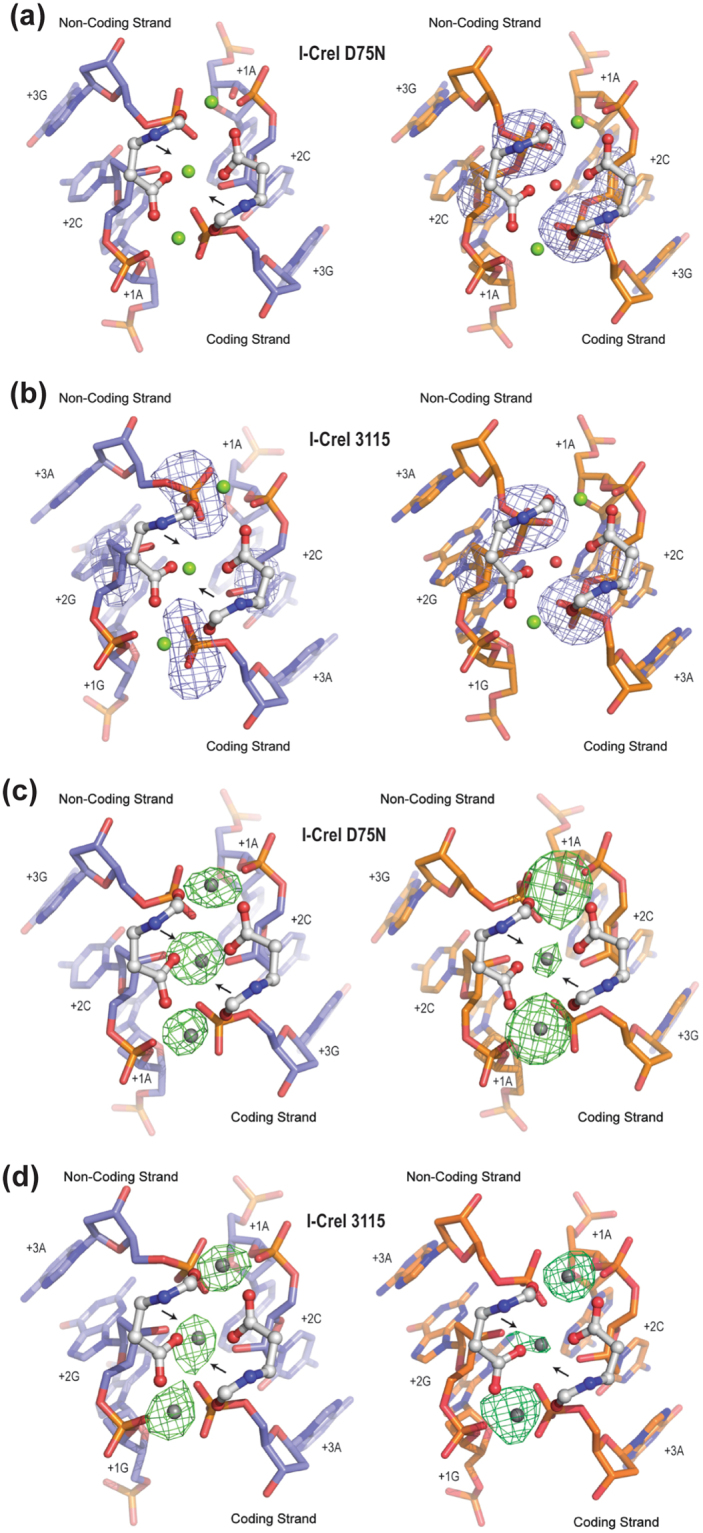
Active site detailed view of both I-CreI variants in complex with cleavable and non-cleavable targets. (**a**) Cleavage analysis of I-CreI_D75N_target (left panel) vs I-CreI_D75N_target-null (right panel) and (**b**) I-CreI_3115_target (left panel) vs I-CreI_3115_target-null (right panel). Omit map (σ = 6.0) around the cleavable phosphodiester revealed no cleavage in the corresponding target-null and phosphodiester cleavage with their original targets. The green spheres represent the Mg^2+^ ions and the red sphere a water molecule. (**c**) Metal positioning analysis of I-CreI_D75N_target (left panel) vs I-CreI_D75N_target-null (right panel) and (**d**) I-CreI_3115_target (left panel) vs I-CreI_3115_target-null (right panel). Anomalous maps (σ = 6.0) around the active site revealed the presence of Mn^2+^ (grey spheres) as a catalytic ion with different occupancies depending on the target to cleave. Black arrows indicate the phosphodiester bond cleaved.

To understand the molecular mechanism that inhibits cleavage when a G is at −4 position flanked by a pyrimidine at position −5, we solved the crystal structures of the cleavable and the corresponding non-cleavable variant targets in the presence of catalytic (Mg^2+^) and non-catalytic (Ca^2+^) cations (Supplementary Table S2, Supplementary Table S4). As observed in our previous studies concerning the central 2NN target region^12^, the absence of cleavage may arise not only from binding differences but also from changes of the DNA structure at the 5NNN region. To check this point, we analysed a superimposition of the crystal structures of both I-CreI variants bound to their target DNA sequences in the non-cleaved state (Supplementary Fig. S3). In both cases subtle protein-DNA interaction changes occurred at the 5NNN region modified, but they did not alter the conformation of the active site, suggesting that cleavage differences were not due to alterations of the catalytic centre configuration. After discarding DNA conformational changes at 5NNN region that could have affected the configuration of the active site, we analysed the crystal structures of both I-CreI_D75N and I-CreI_3115 proteins in complex with cleavable and non-cleavable DNA target sequences and in the presence of the catalytic ion Mg^2+^ in order to decipher the molecular basis of this different behaviour. As expected from *in vitro* cleavage experiments, the comparison of the crystal structures of the I-CreI variants in complex with its cleavable and non-cleavable target DNAs in favourable catalytic conditions (2 mM Mg^2+^), showed the hydrolysed phosphodiesters in the cleavable targets (Fig. 3a,b left panels) while the non-cleavable targets displayed the intact phosphodiester bonds (Fig. 3a,b right panels). Noteworthy, the comparison of the isomorphous signal of the electron density maps of the I-CreI variants, suggested a different number of catalytic ions in the active site for the cleavable and non-cleavable targets, even though the configuration of the active sites was similar. We observed that the structures of the variants in complex with the cleavable targets showed the presence of the 3 reported catalytic ions^15^, while the structures in complex with the non-cleavable targets indicated the presence of just 2 catalytic ions, with the central metal position occupied by a water molecule. The absence of the central metal ion in the non-cleaved structures^16^, would explain the different catalytic behaviour. To assess unambiguously the number of catalytic ions present in the active site, we solved the crystal structure of the two proteins in complex with both target DNAs in the presence of Mn^2+^, which allows catalysis and unambiguous detection and location through its anomalous diffraction signal. In addition, Mn^2+^ is less restrictive than Mg^2+^^17^ allowing phosphoryl transfer reactions in non-ideal substrates^18^, including the non-cleavable targets studied here. In-depth analysis showed differences in the occupancy of the central metal at the active site between cleavable (Fig. 3c,d left panels) and non-cleavable targets (Fig. 3c,d right panels). A high occupancy level of the central metal ion is essential for the cleavage^16^, and the lower occupancy level of the central Mn^2+^ ion found in the non-cleavable targets (46% and 74% for I-CreI_D75N_target-null and I-CreI_3115_target-null, respectively) compared with the cleavable ones (100% in both cases; see Supplementary Table S2) revealed that the non-cleavable targets interfere with the positioning of the central metal ion affecting catalysis.

### Molecular dynamics simulations reveal a link between 5NNN region and key Adenine +1

**Figure 4:**
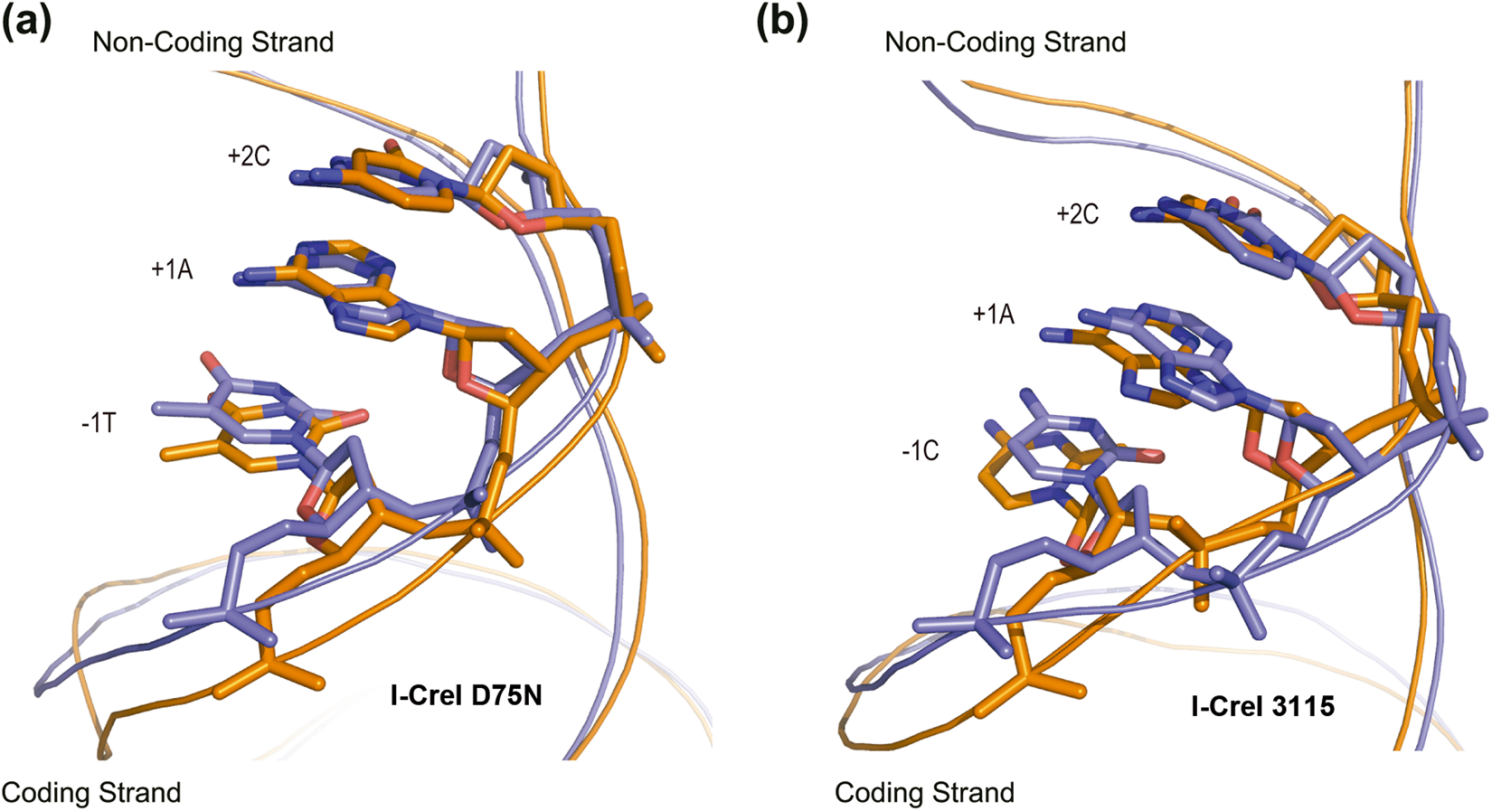
Zoom view at the Adenine +1 remarking the stacking changes between cleavable and non-cleavable targets. (**a**) Structure comparison between I-CreI_D75N_target (blue), where Adenine follows the ideal B-DNA geometry, vs I-CreI_D75N_target-null (orange), where Adenine +1 largely is deviated with respect to the ideal B-DNA geometry. (**b**) Structure comparison between I-CreI_3115_target (blue), where Adenine follows the ideal B-DNA geometry, vs I-CreI_3115_target-null (orange), where Adenine +1 largely is deviated with respect to the ideal B-DNA geometry.

To shed light on possible dynamic-conformational effects involved in the DNA cleavage process, we performed MD simulations of the I-CreI_3115 and I-CreI_D75N bound to the different DNA target sequences (Supplementary Table S3). As reported in our previous works^12,16,19^, the cleavage activity of this class of enzymes can be rationalized in terms of both the proper arrangement of the active site (including water, ions and DNA) and the correct perturbation provided by the protein. Therefore, we compared here the DNA conformational dynamics near the cleavage site. Our results clearly point out that in the cases of I-CreI_D75N_target-null and I-CreI_3115_target-null sequences bound to the I-CreI_D75N and I-CreI_3115 proteins, A at position +1 largely deviates with respect to the usual B-DNA conformation (Fig. 4). In particular, we found that in ~ 30% (I-CreI_D75N_target-null, Fig. 4a) and ~ 60% (I-CreI_3115_target-null, Fig. 4b) of the simulation time A +1 is not paired and is found stacked to the corresponding nucleobases. On the other hand, when the two enzyme variants were simulated bound to the I-CreI_D75N_target and I-CreI_3115_target DNA sequences, the helical parameters of A +1 were similar to those found in the ideal B-DNA conformation, with minor fluctuations typical of atomic thermal motions (Fig. 4a,b). These results suggest that the DNA sequences in I-CreI_D75N_target-null and I-CreI_3115_target-null allow for a larger conformational flexibility to A +1, making the proper positioning of the P-O bond less frequent as compared with the DNA sequence in I-CreI_D75N_target and I-CreI-3115_target. Hence, although in all cases the DNA deviates from the ideal B-DNA conformation, their sequences in the I-CreI_D75N_target-null and I-CreI_3115_target-null are much more flexible, thus probably sampling wider regions of the conformational space not compatible with the phosphorous-oxygen bond break.

## Discussion

The specificity of I-CreI, a widely-redesigned enzyme with therapeutic and biotechnological purposes, strongly depends on indirect readout to recognize and cleave its target sequence. So far, the redesign of the protein-DNA binding properties in this scaffold has been performed taking into account the specific protein-DNA contacts. In this sense, understanding of the 2NN region role in target recognition and cleavage had a strong impact in meganuclease engineering targeting new DNA sequences that avoid the presence of the non-preferred bases in the central region, thus optimizing meganuclease tailoring. Here we provide evidence that the location of a guanine at position −4 (Fig. 1) of the target DNA, in the 5NNN region, strongly affects the conformational dynamics of the adenine at position +1 in the active site independently of the target sequence or protein variant. The larger flexibility of the A +1 in the active centre explains the low occupancy of the central metal ion within the active site, disturbing the positioning of the P-O bond for catalysis, thus inhibiting cleavage. This correlation between bases at −4 and +1 positions of the target depends mainly on DNA sequence composition, thus adding new restrictions to those imposed by the 2NN^12^ (Fig. 1).

Hence, the molecular assembly of the protein-DNA complex is strongly dependent on the DNA sequence in the 5NNN region, regardless of the specific contacts arising from the interaction between the protein and other DNA regions. This suggests the need of a restrictive sequence induced conformation around the active site to allow the formation of a ternary protein-DNA-metal complex competent for cleavage. Our results highlight the importance of the indirect readout of the DNA target in the I-CreI scaffold. The combination of these restrictions in the 2NN and 5NNN regions provide a new scenario for the selection of adequate sequences to be recognized for a given I-CreI variant in genome editing.

## Methods

### Protein expression and purification

The homodimeric I-CreI_D75N variant was cloned and expressed as reported^20^. The co-expression, purification and storage of the heterodimeric I-CreI_3115 variant (Y33G/Q38K/Q44K/R68Y/R70S/D75N/I77Y/I132V-G19S/Y33V/Q38R/S40Q/Q44D/R68A/R70S/D75K/I77R) was carried out as described^13^. Both proteins are folded and share biophysical properties (oligomerization status) with the respective wild type proteins as assessed by circular dichroism and SEC-MALS.

### *In vitro* cleavage assay conditions

Plasmid cleavage assays were performed at 37 °C in 10 mM Tris-HCl (pH 8), 50 mM NaCl, 10 mM MgCl_2_ and 1 mM DTT. 100 nM of the linearized plasmid target (3-Kb) and 120-0.25 nM dilutions of I-CreI_D75N and I-CreI_3115 proteins were used reaching 25 μl of final reaction volume. Reactions were stopped after 1 hour by addition of 5 μl of 45% Glycerol, 95 mM EDTA (pH 8), 1.5% (w/v) SDS, 1.5 mg/ml Proteinase K and 0.048% (w/v) Bromophenol blue (6x Buffer Stop), incubated at 37 °C for 30 minutes and electrophoresed in a 1% agarose gel^20^. The gels were stained with SYBR Safe DNA gel staining kits (Invitrogen) and the intensity of the bands observed upon illumination with UV light was determined with the ImageJ software (http://rsb.info.nih.gov/ij/). The linearized target plasmid was 3 kb in size and yielded two smaller bands, of 2 kb and 1 kb, upon cleavage with the meganuclease. The percentage of cleavage was calculated with the following equation: % cleavage = 100 × (I_2kb_ + I_1kb_)/(I_3kb_ + I_2kb_ + I_1kb_), where I_1kb_, I_2kb_ and I_3kb_ are the intensities of the 1, 2 and 3 kb bands, respectively.

Double-stranded oligonucleotide cleavage assays were performed at 37 °C in 10 mM Tris-HCl (pH 8), 50 mM NaCl, 10 mM MgCl_2_ and 1 mM DTT, in 10 ml of final reaction volume. Concentrations were: 1 mM for the 6 FAM-labelled DNA duplexes (Supplementary Table S3), and 2 μM for I-CreI_D75N and I-CreI_3115 proteins. Reactions were stopped after 30 minutes by addition of 2 μl of 45% Glycerol, 95 mM EDTA (pH 8), 1.5% (w/v) SDS, 1.5 mg/ml Proteinase K and 0.048% (w/v) Bromophenol blue, incubated at 37 °C for 30 minutes and electrophoresed in a 3.5% agarose gel.

The D75N mutation of the I-CreI scaffold does not affect protein structure and facilitates the enzyme purification. As it has been reported previously, I-CreI and its D75N variant display similar *in vitro* activities and levels of specificity^6^.

### Binding Assays by MicroScale Thermophoresis (MST)

MST experiments were performed on a NanoTemper Monolith NT.115 instrument with blue/red channels. Most of the experiments were performed using fluorescently labelled proteins and titrating with the corresponding DNAs, except for the I-CreI_3115/I-CreI_3115_target interaction which was also performed titrating the fluorescently labelled DNA with increasing concentrations of protein. Both labelling approaches gave similar results (Supplementary Table S1). Cy5-labeled I-CreI_3115_target oligo-nucleotide (coding strand) was purchased from TAG Copenhagen A/S (Copenhagen, Denmark) and annealed with its complementary oligonucleotide prior to MST experiments. I-CreI_3115 and I-CreI_D75N were labelled using the Monolith NT Protein Labelling Kit RED-NHS (NanoTemper Technologies GmbH) according to the supplied protocol. The fluorophore-conjugated protein and the extent of labelling were determined by spectrophotometry. MST experiments were performed at 25 °C in 50 mM Tris pH 8.0, 300 mM NaCl, 2 or 10 mM CaCl_2_, 0.5 mM TCEP, 0.05% Tween-20 (in the case of I-CreI_3115) or in 50 mM Hepes pH 7.5, 300 mM NaCl, 10 mM CaCl_2_, 0.5 mM TCEP, 0.05% Tween-20 for the I-CreI_D75N protein. In all cases, premium coated capillaries were used and measurements were performed at 40% MST power. Laser off/on times were 5 s and 30 s, respectively. The fluorescently labelled DNA or proteins were used at 25 nM concentration. The fluorescently labelled partner was mixed with equivalent volumes of a two-fold serial dilution of the different ligands starting at concentrations of 16 μM or 512 μM (for the non-cleavable DNA variants). The fraction of bound protein was derived from the ratio of the averaged normalized fluorescence at later (2.0 s prior to switching off the IR-laser) and early times (0.5 s after switching on the IR-laser) of the thermophoretic time traces at different ligand concentrations. The *K*_D_s were obtained by fitting the fraction of bound protein to the quadratic solution of the binding reaction equilibrium derived from the law of mass action. The number of independent repeats was 3 for all measurements.

### Crystallization

The 24-mer DNA oligonucleotides were purchased from Proligo and sequences are described at Supplementary Table S3. The DNA substrates were generated after mixture of equimolar amounts of oligonucleotides in 10 mM Tris-HCl pH 8. All of them form a 24-bp blunt-ended duplexes after incubation for 5 minutes at 99 °C and slowly cooling down to 25 °C, with the reverse complementary oligonucleotide. I-Cre_D75N_target is palindromic and thus self-annealing. The protein–DNA complexes were: I-CreI_D75N:I-CreI_D75N_target:Ca^2+^, I-CreI_D75N:I-CreI_D75N_target:Mn^2+^, I-CreI_D75N:I-CreI_D75N_target:Mg^2+^, I-CreI_D75N:I-CreI_D75N_target-null:Mn^2+^, I-CreI_D75N:I-CreI_D75N_target-null:Mg^2+^, I-CreI_3115:I-CreI_3115_target:Ca^2+^, I-CreI_3115:I-CreI_3115_target:Mn^2+^, I-CreI_3115:I-CreI_3115_target-3115:Mg^2+^, I-CreI_3115:I-CreI_3115_target-null:Mn^2+^, I-CreI_3115:I-CreI_3115_target-null:Mg^2+^ obtained in the presence of 2 mM metal ion. Complexes were formed by pre-warming the meganuclease and the oligonucleotide samples at 37 °C and mixing them in a 0.75:1 molar ratio (DNA:protein). The mixture was incubated for 50 minutes at this temperature, and then spun down for 5 minutes to remove insoluble material. The final concentration of protein in the DNA–protein complex solution was 4 mg/ml.

All protein-DNA complex crystals were grown in the presence of 2 mM Ca^2+^, Mg^2+^ or Mn^2+^ using the hanging-drop method at 290 K, in 2 μl droplets formed by 1 μl of the DNA–protein complex and 1 μl of precipitant solution consisting of 0.05–0.1 M calcium acetate, 0.1 M sodium acetate pH 4.6–5.4, 33–40% (v/v) 1,2-propanediol. The complexes were cryo-protected by adding 20% (v/v) glycerol to the mother liquor before flash-frozen in liquid nitrogen.

### Data collection, structure solution, model building and refinement

All data were collected from frozen crystals at 100 K with PILATUS detectors at beamlines PXI (SLS, Villigen, Switzerland) and XALOC (ALBA Synchrotron, Barcelona, Spain). Data processing and scaling were accomplished with XDS^21^ and Scala from the CCP4 package^22^. Statistics for the crystallographic data and structure solution are summarized in Supplementary Table S2. The structures were solved by molecular replacement, as implemented in the program PHASER^23^. The search models were based on the PDB entries 1G9Y (I-CreI:DNA:Ca^2+^) and 1G9Z (I-CreI:DNA:Mg^2+^). The models were then subjected to iterative cycles of model building and refinement with Coot^24^ and PHENIX^25^. The identification and analysis of the protein–DNA hydrogen bonds and van der Waals contacts was done with the Protein Interfaces, Surfaces and Assemblies service PISA at the European Bioinformatics Institute (http://www.ebi.ac.uk/msdsrv/prot_int/pistart.html). DNA structures were analysed using 3DNA^26^.

### Molecular Dynamics Simulations

The molecular dynamics (MD) simulations lasting 100 ns each were performed using the Gromacs software v 5.0, starting from the experimental crystal structures using the non-cleaved constructs and properly substituting the DNA sequence. We utilized the amber99sb force field^27^ for the simulation of the four systems (I-CreI_D75N and I-CreI_3115 variants bound to their DNA targets and to non-cleavable DNA sequences (null): I-CreI_D75N:I-CreI_D75N_target, I-CreI_D75N:I-CreI_D75N_target-null, I-CreI_3115:I-CreI_3115_target, I-CreI_3115:I-CreI_3115_target-null). The systems were solvated using the SPC water model. After usual minimization, thermalization and equilibration steps, the systems were simulated using an integration step of 2 fs with periodic boundary conditions and Particle Mesh Ewald method for the calculation of the electrostatics. The pressure and temperature were kept constant by means of the Parrinello-Rahman barostat^28^. (P = 1 bar) and the isokinetic temperature coupling algorithm (T = 310 K, the same temperature of the experimental cleavage assays).

### Data availability

The datasets generated during and/or analysed during the current study are available from the corresponding author on reasonable request.

## Electronic supplementary material

Supplementary Information
