# Supplementary material for: Understanding the indirect DNA read-out specificity of I-CreI Meganuclease
Source: Sci Rep. 2018 Jul 6;8:10286. doi: 10.1038/s41598-018-28599-0 (PMC6035192; doi:10.1038/s41598-018-28599-0)
Supplement: Supplementary file 1 — Supplementary Information [file 41598_2018_28599_MOESM1_ESM.pdf]

SUPPLEMENTARY INFORMATION

Understanding the indirect DNA read-out specificity of I-CreI Meganuclease

Jesús Prieto[1], Pilar Redondo[2], Blanca López-Méndez[3], Marco D'Abramo[4], Nekane Merino[5], Francisco J Blanco[5,6], Phillipe Duchateau[7], Guillermo Montoya[3], Rafael Molina[3,*]

[1] Structural Biology and Biocomputing Programme, Spanish National Cancer Research Centre (CNIO), Macromolecular Crystallography Group, c/Melchor Fdez. Almagro 3, 28029 Madrid, Spain

[2] Structural Biology and Biocomputing Programme, Spanish National Cancer Research Centre (CNIO), Cell Signalling and Adhesion Group, c/Melchor Fdez. Almagro 3, 28029 Madrid, Spain

[3] Protein Structure & Function Programme, Novo Nordisk Foundation Center for Protein Research, Faculty of Health and Medical Sciences, University of Copenhagen, Blegdamsvej 3B, 2200 Copenhagen, Denmark.

[4] Department of Chemistry, University of Rome "La Sapienza," Piazzale Aldo Moro 5, 00185, Rome, Italy.

[5] CIC bioGUNE, Parque Tecnológico de Bizkaia Edificio 800, 48160 Derio, Spain.

[6] IKERBASQUE, Basque Foundation for Science, María Díaz de Haro 3, 48013 Bilbao, Spain.

[7] CELLECTIS S.A., 8 rue de la Croix Jarry, 75013 Paris, France

* To whom correspondence should be addressed. Email: rafael.molina@cpr.ku.dk

Keywords: Gene targeting, genetics, protein-DNA interaction, homing endonucleases, X-ray crystallography

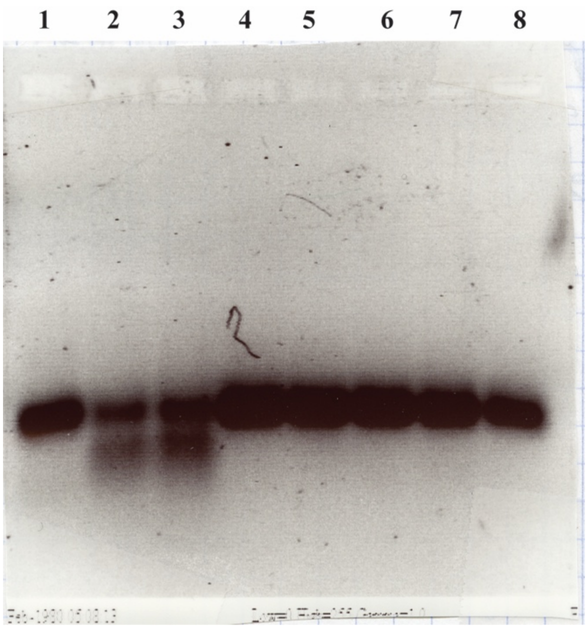

Supplementary Figure S1. Full-length gel from the I-CreI *in vitro* cleavage experiments to validate 5NNN non-cleavable target patterns using labelled duplex DNA targets. Lanes: **1**, I-CreI_D75N_target; **2**, I-CreI_D75N_target in the presence of I-CreI_D75N protein; **3**, I-CreI_3115_target in the presence of I-CreI_3115 protein; **4**, I-CreI_3115_target; **5**, I-CreI_D75N_target-null (non-cleavable derivative); **6**, I-CreI_D75N_target-null in the presence of I-CreI_D75N protein; **7**, I-CreI_3115_target-null (non-cleavable derivative) in the presence of I-CreI_3115 protein; **8**, I-CreI_3115_target-null (non-cleavable derivative).

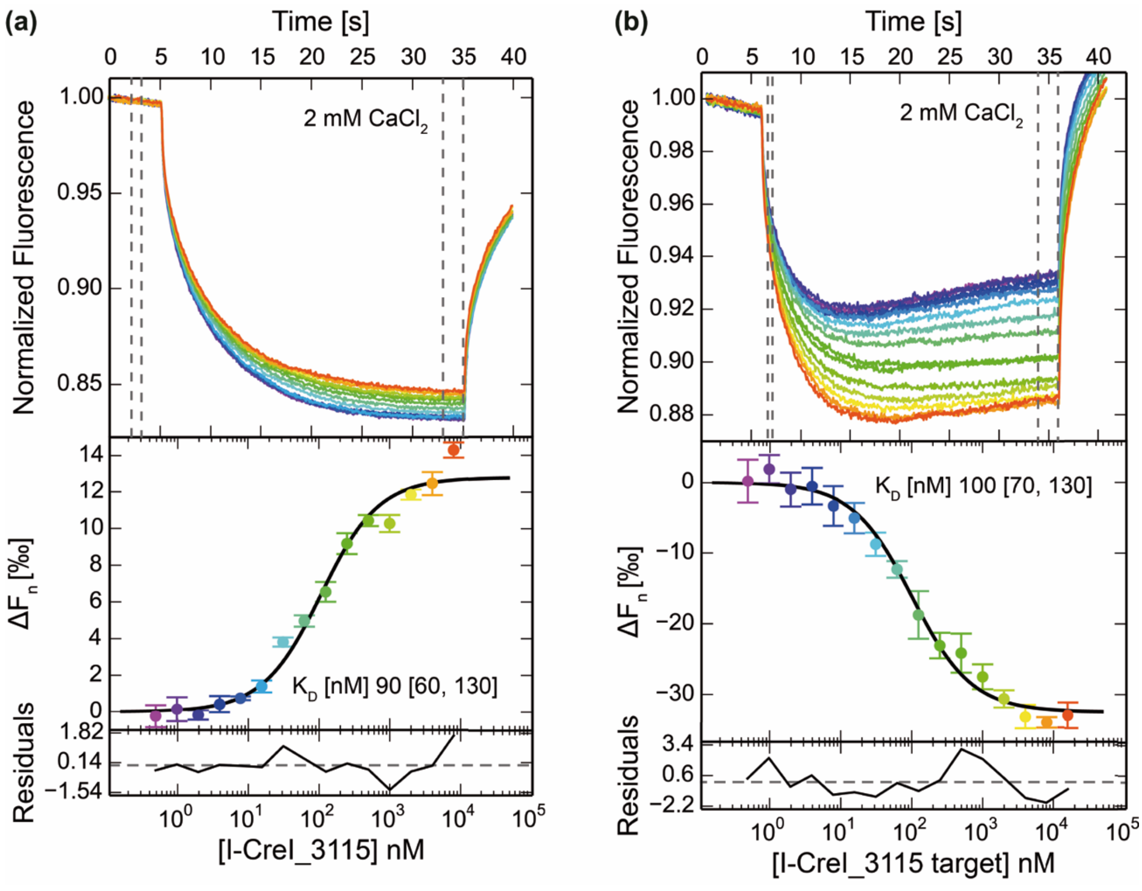

Supplementary Figure S2. Thermophoresis data for the I-CreI_3115/DNAs interactions in the presence of 2 mM $CaCl_2$. I-CreI_3115 and DNA target interaction using complementary labelling schemes, i.e., fluorescently labeled DNA duplex in **(a)** or fluorescently labeled protein in **(b)**. The upper panel shows the thermophoresis time traces with the discontinuous lines boundary regions used to calculate the observed thermophoresis related to the free- and bound-biomolecule populations. The fluorescence is normalized to a starting value of 1.0. The binding curve data is shown in the middle panel, each point is the average of three experiments and the error bars show the standard deviation. The line is the best fit to a 1:1 binding model. The bottom panel shows residuals between the fit and experimental data.

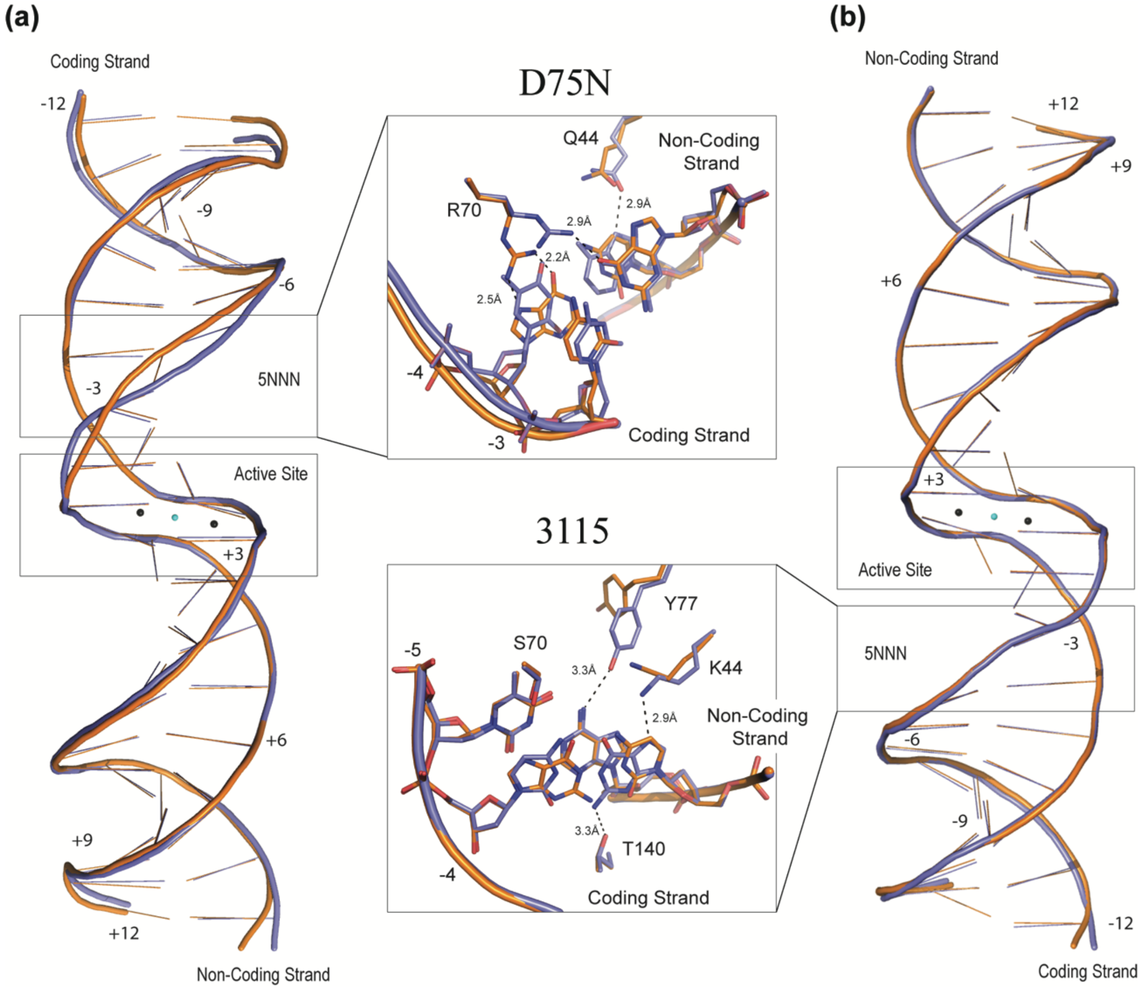

Supplementary Figure S3. DNA structure superimposition of cleavable (blue) and their corresponding non-cleavable (orange) DNA targets in their non-cleavage states including zoom views at the 5NNN region. **(a)** I-CreI_D75N_target vs I-CreI_D75N_target-null DNA target. **(b)** I-CreI_3115_target vs I-CreI_3115_target-null DNA target. The black spheres represent the metal ions and the blue sphere a water molecule.

Supplementary Table S1. *Meganuclease binding affinity for DNA targets measured by microscale thermophoresis.* [a]Cy5-labelled I-CreI-3115 DNA was used at a 25 nM concentration. [b,c]I-CreI_3115 and I-CreI_D75N were fluorescently labelled and used at a 25 nM concentration. [d]Numbers in brackets represent the 68.3% confidence interval for the fitted K_Ds calculated by ESP (error-surface projection).

Fluorescent labelled partner	Ligand	K_D [nM]	$CaCl_2$ [mM]
I-CreI_3115_target[a]	I-CreI_3115	90 [60, 130][d]	2
I-CreI_3115_target[a]	I-CreI_3115	110 [70, 160][d]	10
I-CreI_3115[b]	I-CreI_3115_target	100 [70, 130][d]	2
I-CreI_3115[b]	I-CreI_3115_target	100 [60, 160][d]	10
I-CreI_3115[b]	I-CreI_3115_target-null	500 [200, 1200][d] x 10^3	2
I-CreI_3115[b]	I-CreI_3115_target-null	260 [110, 750][d] x 10^3	10
I-CreI_D75N[c]	I-CreI_D75N_target	120 [90, 180][d]	10
I-CreI_D75N[c]	I-CreI_D75N_target-null	33 [24, 48][d] x 10^3	10

Supplementary Table S2. *Data collection and refinement statistics.*

	I-CreI3115: I-CreI_ta-3115: Ca^{2+}	I-CreI3115: I-CreI_ta-3115: Mg^{2+}	I-CreI3115: I-CreI_ta-3115: Mn^{2+}
**PDB code**	6FB0	6FB1	6FB2
**Data collection**			
Space group	$P2_1$	$P2_1$	$P2_1$
Cell dimensions			
a, *b*, *c* (Å)	45.44, 67.67, 83.53	45.10, 66.66, 91.39	44.89, 67.36, 90.87
α, β, γ (°)	90, 96.75, 90	90, 96.80, 90	90, 96.80, 90
Wavelength	0.87	1.00	1.60
Resolution (Å)	37.81-2.15 (2.27-2.15)*	66.66-3.00 (3.15-3.00)*	44.57-2.95 (3.11-2.95)*
R_{merge}	0.07 (0.46)	0.08 (0.47)	0.08 (0.50)
Mean I / σ*I*	10.5 (3.0)	5.4 (1.6)	9.4 (2.3)
Completeness (%)	100 (99.9)	99.7 (98.4)	97.9 (96.6)
Redundancy	4.2 (4.2)	2.4 (2.4)	3.5 (3.4)
**Refinement**			
A site metal occupancy	100 (Ca^{2+})	100 (Mg^{2+})	100 (Mn^{2+})
B site metal occupancy	100 (Ca^{2+})	100 (Mg^{2+})	100 (Mn^{2+})
C site metal occupancy	0	100 (Mg^{2+})	100 (Mn^{2+})
Resolution (Å)	33.84-2.15	33.98-3.02	41.99-2.95
No. Reflections	26697	18027	11150
R_{work} / R_{free}	0.18/0.23	0.21/0.25	0.19/0.23
No. Molecules in a.u.	1	1	1
DNA strands cleaved?	None	Both	Both
No. Ions at a.s.	2	3	3
No. Atoms			
Protein	2486	2486	2486
DNA	978	980	980
Ions	4	3	3
Water	180	15	11
R.m.s. deviations			
Bond lengths (Å)	0.013	0.002	0.003
Bond angles (°)	1.154	0.696	0.803

*Values in parentheses are for highest-resolution shell. One crystal was used to solve each structure.

Supplementary Table S2 (Cont.). *Crystallographic data collection and refinement statistics.*

	I-CreI3115: I-CreI_ta-3115-null: Mg^{2+}	I-CreI3115: I-CreI_ta-3115-null: Mn^{2+}	I-CreI: I-CreI_ta: Ca^{2+}
**PDB code**	6FB5	6FB6	4AAG
**Data collection**			
Space group	$P2_1$	$P2_1$	$P22_12_1$
Cell dimensions			
a, *b*, *c* (Å)	45.29, 68.00, 83.03	45.18, 67.99, 83.04	47.47, 71.41, 171.21
α, β, γ (°)	90, 96.55, 90	90, 96.45, 90	90, 90, 90
Wavelength	1.00	1.60	1.00
Resolution (Å)	41.24-2.20 (2.32-2.20)*	44.89-2.60 (2.74-2.60)*	47.47-2.80 (2.95-2.80)*
R_{merge}	0.07 (0.25)	0.11 (0.54)	0.09 (0.48)
Mean I / σ*I*	8.9 (3.4)	10.6 (3.3)	5.3 (1.5)
Completeness (%)	99.5 (96.7)	96.1 (95.6)	99.9 (99.7)
Redundancy	3.3 (3.0)	6.8 (6.7)	4.2 (3.9)
**Refinement**			
A site metal occupancy	100 (Mg^{2+})	100 (Mn^{2+})	100 (Ca^{2+})
B site metal occupancy	100 (Mg^{2+})	100 (Mn^{2+})	100 (Ca^{2+})
C site metal occupancy	0	74 (Mn^{2+})	0
Resolution (Å)	37.73-2.20	44.89-2.60	45.74-2.80
No. Reflections	47710	28700	14934
R_{work} / R_{free}	0.19/0.22	0.19/0.24	0.18/0.25
No. Molecules in a.u.	1	1	1
DNA strands cleaved?	None	Both	None
No. Ions at a.s.	2	3	2
No. Atoms			
Protein	2477	2477	2459
DNA	978	981	977
Ions	4	3	2
Water	127	11	40
R.m.s. deviations			
Bond lengths (Å)	0.021	0.003	0.008
Bond angles (°)	0.821	0.780	1.280

*Values in parentheses are for highest-resolution shell. One crystal was used to solve each structure.

Supplementary Table S2 (Cont.). *Crystallographic data collection and refinement statistics.*

	I-CreI: I-CreI_ta: Mg^{2+}	I-CreI: I-CreI_ta: Mn^{2+}	I-CreI: I-CreI_ta-null: Mg^{2+}
**PDB code**	4AAB	6FB7	6FB8
**Data collection**			
Space group	$P22_12_1$	$P22_12_1$	$P4_3$
Cell dimensions			
a, *b*, *c* (Å)	46.81, 70.93, 171.51	49.02, 71.50, 169.15	79.48, 79.48, 125.76
α, β, γ (°)	90, 90, 90	90, 90, 90	90, 90, 90
Wavelength	1.00	1.60	1.00
Resolution (Å)	44.51-2.50 (2.64-2.50)*	49.02-2.69 (2.83-2.69)*	27.44-2.45 (2.58-2.45)*
R_{merge}	0.07 (0.41)	0.10 (0.55)	0.11 (0.64)
Mean $I / \sigma I$	9.9 (1.9)	15.1 (3.7)	8.4 (2.1)
Completeness (%)	99.9 (100)	97.1 (93.5)	100.0 (99.9)
Redundancy	4.8 (4.9)	8.8 (8.6)	6.1 (6.0)
**Refinement**			
A site metal occupancy	100 (Mg^{2+})	100 (Mn^{2+})	100 (Mg^{2+})
B site metal occupancy	100 (Mg^{2+})	100 (Mn^{2+})	100 (Mg^{2+})
C site metal occupancy	100 (Mg^{2+})	100 (Mn^{2+})	0
Resolution (Å)	34.82-2.50	49.02-2.69	27.42-2.45
No. Reflections	20614	30910	56079
R_{work} / R_{free}	0.17/0.22	0.17/0.21	0.19/0.22
No. Molecules in a.u.	1	1	1
DNA strands cleaved?	Both	Both	None
No. Ions at a.s.	3	3	2
No. Atoms			
Protein	2472	2472	2495
DNA	979	979	978
Ions	3	3	4
Water	122	22	87
R.m.s. deviations			
Bond lengths (Å)	0.007	0.009	0.010
Bond angles (°)	1.234	0.992	1.263

*Values in parentheses are for highest-resolution shell. One crystal was used to solve each structure.

Supplementary Table S2 (Cont.). *Crystallographic data collection and refinement statistics.*

	I-CreI: I-CreI_ta-null: Mn^{2+}
**PDB code**	6FB9
**Data collection**	
Space group	$P4_3$
Cell dimensions	
a, *b*, *c* (Å)	79.73, 79.73, 126.25
α, β, γ (°)	90, 90, 90
Wavelength	1.80
Resolution (Å)	49.49-2.95 (3.11-2.95)*
R_{merge}	0.08 (0.56)
Mean I / σ*I*	12.4 (2.3)
Completeness (%)	98.6 (97.6)
Redundancy	3.8 (3.7)
**Refinement**	
A site metal occupancy	100 (Mn^{2+})
B site metal occupancy	100 (Mn^{2+})
C site metal occupancy	46 (Mn^{2+})
Resolution (Å)	42.05-2.95
No. Reflections	32279
R_{work} / R_{free}	0.17/0.20
No. Molecules in a.u.	1
DNA strands cleaved?	Both
No. Ions at a.s.	3
No. Atoms	
Protein	2487
DNA	980
Ions	5
Water	22
R.m.s. deviations	
Bond lengths (Å)	0.010
Bond angles (°)	1.287

*Values in parentheses are for highest-resolution shell. One crystal was used to solve each structure.

Supplementary Table S3. *Oligonucleotide sequences sampled as I-CreI DNA target* (labelled at 5' for in vitro cleavage assays / MST experiments and not labelled for crystallization experiments). Bold letters represent oligonucleotide changes derived from their corresponding wild-type DNA sequence.

Number	DNA target	Sequence
1	I-CreI D75N wild-type	5'-TCAAAACGTCGTACGACGTTTTGA-3'
2	I-CreI D75N null	5'-TCAAAAC**TG**CGTACGACGTTTTGA-3'
3	I-CreI 3115 wild-type	5'-TCAGACTTCTCCACAGGAGTCAGA-3'
4	I-CreI 3115 null	5'-TCAGACTT**G**TCCACAGGAGTCAGA-3'

Supplementary Table S4. *Summary of crystallographic data.*
In light green are the structures utilized in the molecular dynamics simulations.

**Structure**	**Metal Concentration**	**Number**	**Cleaved**
I-CreI_D75N: I-CreI_D75N_target	Ca^{2+} [2 mM]	2	No
I-CreI_D75N: I-CreI_D75N_target	Mn^{2+} [2 mM]	3	Yes
I-CreI_D75N: I-CreI_D75N_target	Mg^{2+} [2 mM]	3	Yes
I-CreI_D75N: I-CreI_D75N_target-null	Mn^{2+} [2 mM]	3	Yes
I-CreI_D75N: I-CreI_D75N_target-null	Mg^{2+} [2 mM]	2	No
I-CreI_3115: I-CreI_3115_target	Ca^{2+} [2 mM]	2	No
I-CreI_3115: I-CreI_3115_target	Mn^{2+} [2 mM]	3	Yes
I-CreI_3115: I-CreI_3115_target	Mg^{2+} [2 mM]	3	Yes
I-CreI_3115: I-CreI_3115_target-null	Mn^{2+} [2 mM]	3	Yes
I-CreI_3115: I-CreI_3115_target-null	Mg^{2+} [2 mM]	2	No
